# The effectiveness of pre-contoured titanium alloy rods in inducing thoracic kyphosis after sequential spinal releases in an *in vitro* biomechanical model

**DOI:** 10.3389/fsurg.2023.1064037

**Published:** 2023-05-03

**Authors:** Jinhui Shi, Nathaniel R. Ordway, Mike H. Sun, Stephen A. Albanese, William F. Lavelle

**Affiliations:** SUNY Upstate Medical University, Department of Orthopedic Surgery, Syracuse, NY, United States

**Keywords:** thoracic kyphosis, posterior release, pre-contoured rod, adolescent idiopathic scoliosis, spinal deformity

## Abstract

**Purpose:**

Evaluate the ability of pre-contoured rods to induce thoracic kyphosis (TK) in human cadaveric spines and determine the effectiveness of sequential surgical adolescent idiopathic scoliosis (AIS) release procedures.

**Methods:**

Six thoracolumbar (T3-L2) spine specimens were instrumented with pedicle screws bilaterally (T4–T12). Over correction using pre-contoured rods was performed for intact condition and Cobb angle was measured. Rod radius of curvature (RoC) was measured pre- and post-reduction. The process was repeated following sequential release procedures of (1) interspinous and supraspinous ligaments (ISL); (2) ligamentum flavum; (3) Ponte osteotomy; (4) posterior longitudinal ligament (PLL); and (5) transforaminal discectomy. Cobb measurements determined the effective contribution of release on TK and RoC data displayed effects of reduction to the rods.

**Results:**

The intact TK (T4–12) was 38.0° and increased to 51.7° with rod reduction and over correction. Each release resulted in 5°–7°of additional kyphosis; the largest releases were ISL and PLL. All releases resulted in significant increases in kyphosis compared to intact with rod reduction and over correction. Regionally, kyphosis increased ∼2° for each region following successive releases. Comparing RoC before and after reduction showed significant 6° loss in rod curvature independent of release type.

**Conclusion:**

Kyphosis increased in the thoracic spine using pre-contoured and over corrected rods. Subsequent posterior releases provided a substantial, meaningful clinical change in the ability to induce additional kyphosis. Regardless of the number of releases, the ability of the rods to induce and over correct kyphosis was reduced following reduction.

## 1. Introduction

Spinal deformities associated with adolescent idiopathic scoliosis (AIS) have historically been thought of as a coronal plane issue. Deformity techniques have concentrated on restoring the coronal plane alignment. More recently, surgeons have begun to understand the importance of the sagittal plane (1). Reconstructive procedures that do not involve restoration of lumbar lordosis have been found to be detrimental (2–4). Failure to restore lumbar lordosis may result in sagittal plane imbalance with the associated disability of a flat back deformity. Reconstruction of normal thoracic kyphosis is also potentially important. A patient's thoracic kyphosis is considered the primary sagittal curve whereby an individual develops their secondary cervical lordosis and lumbar lordosis (5). The implications for failure to restore normal thoracic kyphosis in the treatment of adolescent idiopathic scoliosis are just beginning to be understood (6, 7).

Newton et al. (8) evaluated the sagittal alignment of thoracic AIS using EOS as a “3-D sagittal plane”, and the results showed that thoracic AIS patients had considerable thoracic hypokyphosis prior to surgery. Sullivan et al. (9) concluded the increasing severity of coronal plane curvature was associated with a progressive loss of thoracic kyphosis, and the appropriate intraoperative techniques for correction of idiopathic scoliosis should be applied in all three planes. Unfortunately, several studies (10, 11) have indicated that posterior pedicle screw correction and fixation are not efficient in restoring thoracic kyphosis to a normal range. Surgeons have come to better understand the sagittal balance of the global spine. In an attempt to improve sagittal plane reconstruction, surgeons may choose to pre-contour the rods to induce thoracic kyphosis; however, this is often limited by the rigidity of the spine (12). Numerous techniques for releasing the spine have been described (13–15). These includes ligamentous releases, discectomy procedures as well as bony resections.

To our knowledge, the extent to which these releases improve the ability for a contoured rod to induce thoracic kyphosis has not been described. The purpose of this study was to evaluate the ability of pre-contoured rods to induce thoracic kyphosis in a cadaveric spine and to determine the effectiveness sequential posterior releasing procedures.

## 2. Methods

### 2.1. Specimen preparation

Fresh thoracolumbar spine specimens, T3-L2, were dissected from six cadavers (4 females, 2 males; age range: 24–66 years). The specimens were chosen based on the following inclusion criteria: no prior spinal surgery or instrumentation, free of osteophytes, no significant degeneration (disc height collapse), no spinal deformity or osteoporosis. Specimens were manually manipulated to ensure reasonable flexibility in all levels of interest. Lateral and anterior-posterior (AP) radiographs were acquired to confirm all levels were free of spinal deformity and did not have significant degeneration or other pathological conditions. Dual energy x-ray absorptiometry (DEXA) scans were performed on a Lunar DPX-IQ Pencil Beam Densitometer (General Electric, Louisville, KY) and bone mineral density (BMD) measurements were acquired using a lumbar protocol. Using the World Health Organization (WHO) definition for osteopenia, the *T*-score had to be greater than −1.5 to be included in the study.

Monoaxial pedicle screws (Expedium, Depuy Spine, Raynham, MA) of an appropriate diameter for each pedicle (approximately 80% fill) were placed from T4–T12 bilaterally using a free hand technique based on anatomic landmarks. The screw placement was confirmed by direct palpation of the screw path with a ball tip probe as well as a visual inspection with a C-arm.

### 2.2. Spinal releases examined

All specimens were examined initially with a Cobb angle measurement from T4–12 from a lateral radiograph. A rod contouring and reduction process followed as well as a subsequent, sequential release process (see subsequent paragraph). Each thoracolumbar spine specimen was tested independently from intact to final release. Surgical releases were performed from T4–T12, and each Release condition was with instrumentation applied:

Intact – Uninstrumented thoracic spine specimen.
Release 0 – Intact specimen, no ligamentous releases or osteotomy.
Release 1 – The intraspinous and supraspinous ligaments (ISL) were transected.
Release 2 – The ligamentum flavum was transected.
Release 3 – The facet joints were removed with Kerrison's consistent with a Ponte osteotomy.
Release 4 – The posterior longitudinal ligament (PLL) was transected.
Release 5 – The discectomy was performed from a transforaminal approach.

### 2.3. Rod contouring and reduction

**Figure 1:**
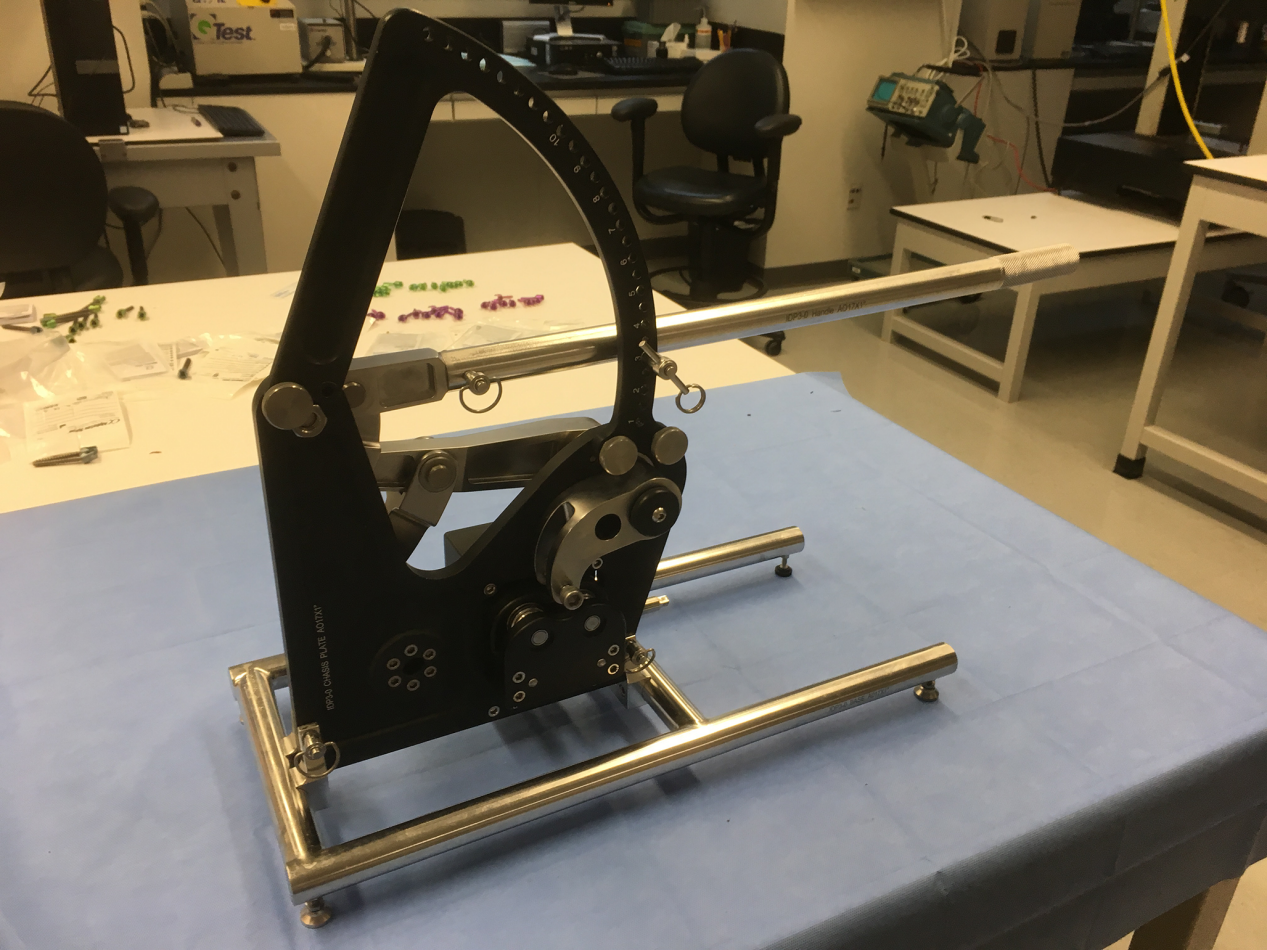
A novel automated rod bender was used to create consistent, over corrected contour based upon the level of kyphosis.

**Figure 2:**
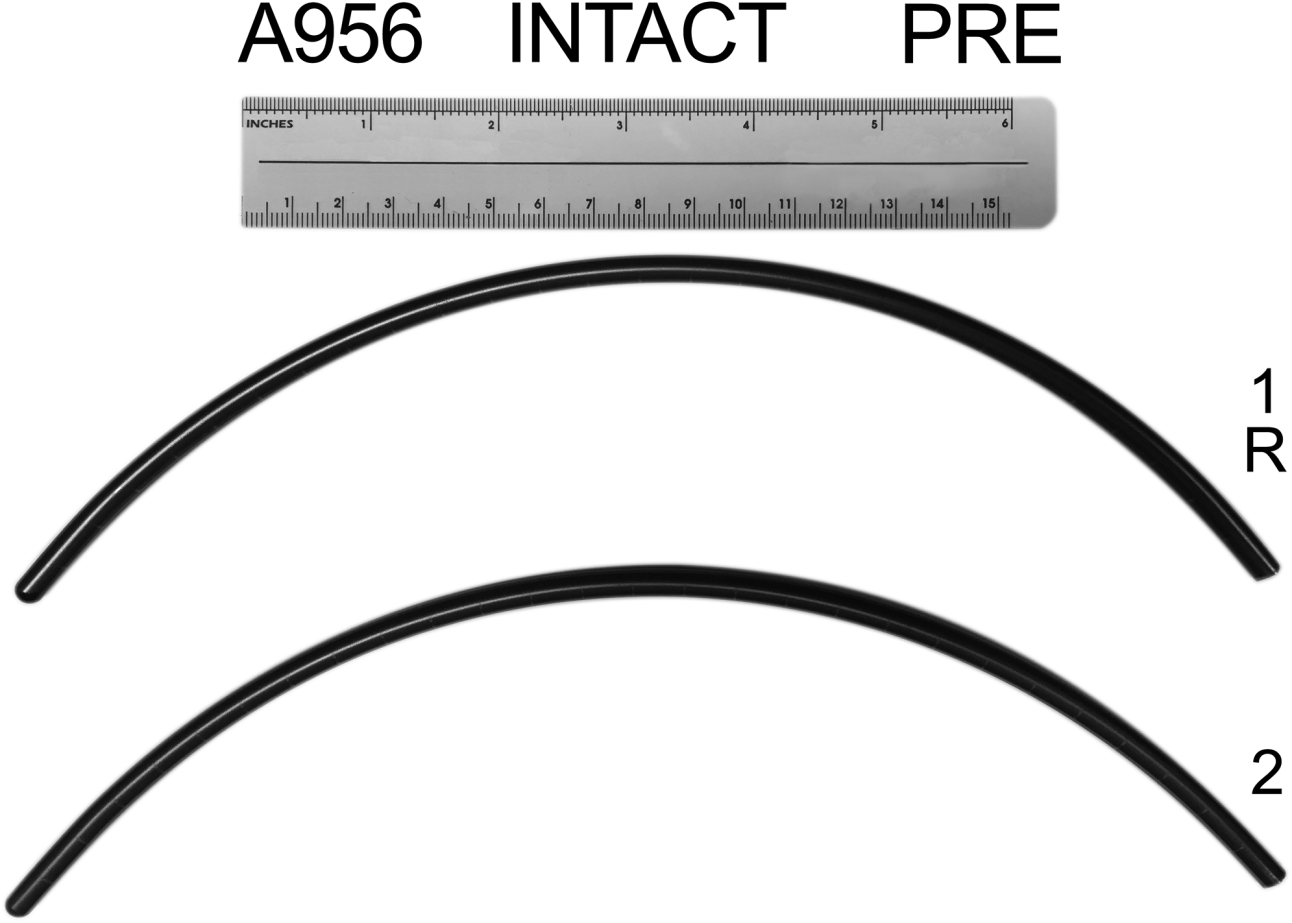
An example image of bilateral, contoured spinal rods prepared for reduction and correction process. The chord length and height are used to calculate the radius of curvature (RoC) for each rod.

In addition to the initial intact Cobb measurement, Cobb angle from T4–12 was determined for each Release condition. A novel rod-bender (Figure 1) was used to create symmetrically bent rods at a variety of curvatures. This allowed generation of consistent, over corrected contoured rods based on the Cobb angle. A new pair of 5.5 mm titanium alloy rods was utilized for each thoracolumbar spine specimen for the kyphotic correction procedure. The lengths of each pair of rods were cut based on the length of T4–T12 while allowing extra length in consideration of the forthcoming 5 releases and overcorrection at each step. The rod radius of curvature (RoC) was determined prior to the application to confirm the overcorrection and for follow-up data analyses. The rod RoC was determined by quantitatively analyzing digital images (Image J software, ver. 1.52q, NIH) of the rods (Figure 2) and applying the formula:

$$\mathrm{RoC}=\tan^{-1}\left(\frac{2r}{c}\right)$$

In the formula, “*c*” represents the chord length between the ends of the rod and “*r*” is the perpendicular distance from the middle of the cord to the mid arc of the rod. Incremental changes in rod RoC were performed with the rod bender to prepare the pair of rods for the subsequent release condition.

At each Release condition, the rods were aligned and placed into the cranial and caudal pedicle screws with loose set caps and then gradually reduced into the apex of the construct utilizing turn down type reduction devices. A lateral radiograph with instrumentation and following the reduction was collected and the Cobb angle measured. The rods were removed, and digital images were collected again so the rod RoC could be determined post-reduction. The process was repeated for each Release condition and with over correcting the rod based on the prior release Cobb angle.

### 2.4. Radiographic measurements

**Figure 3:**
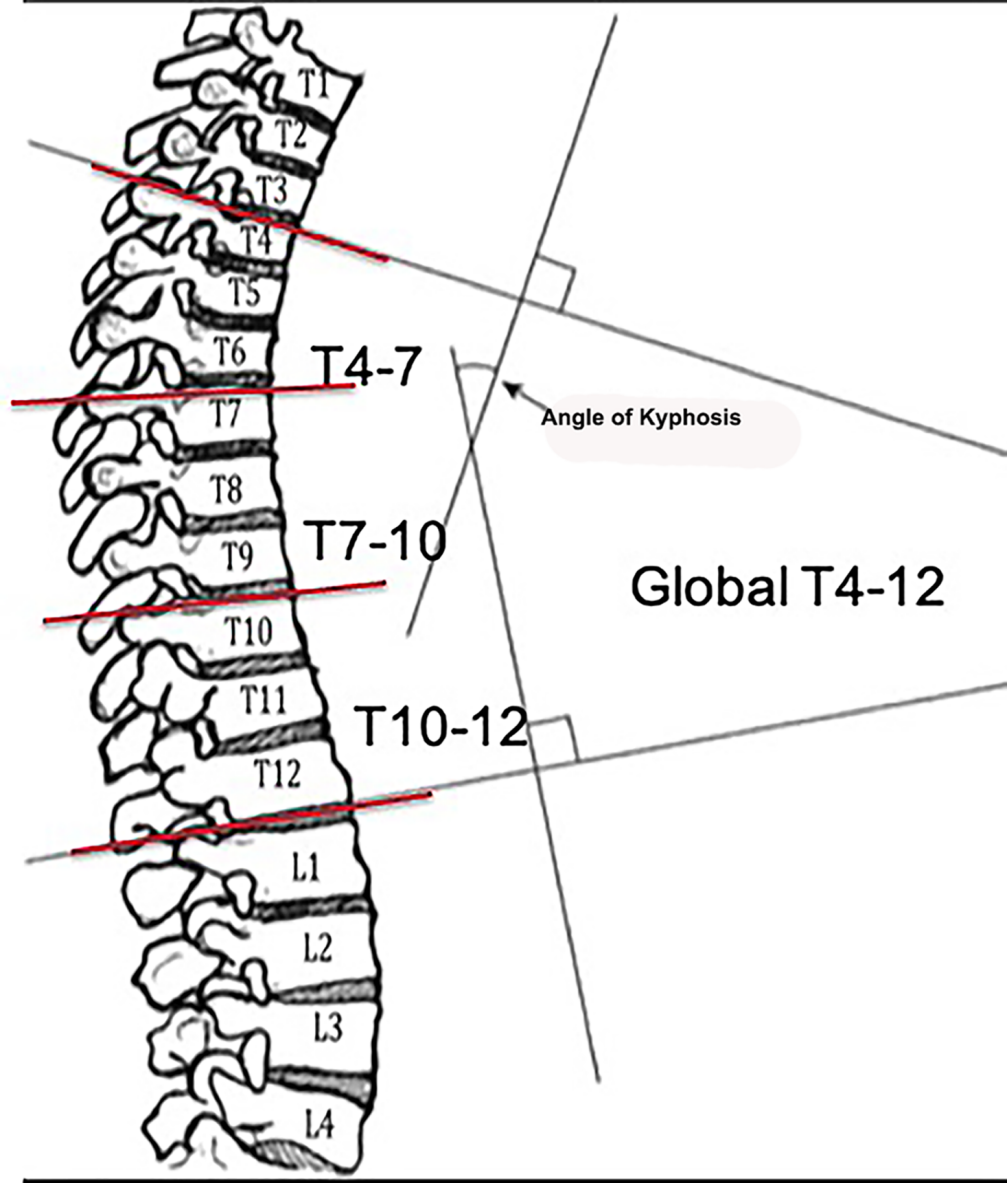
Global and regional thoracic kyphosis measurements collected during the sequential spinal releases.

Cobb angles from the lateral radiographic images were analyzed for all release conditions. The global thoracic kyphosis (TK) was measured from the upper endplate of T4 to the lower endplate of T12. Regional kyphosis was also measured. The superior TK was defined from the upper endplate of T4 to the upper endplate of T7; the middle TK was defined from the upper endplate of T7 to the upper endplate of T10, and the inferior TK which defined from the upper endplate of T10 to the lower endplate of T12. A representation of the global and regional thoracic kyphosis measurements is shown in Figure 3.

### 2.5. Statistical analysis

Descriptive statistics were determined for the global and regional TK measures for the intact and all release conditions (Release 0–5). To examine the effect of Release condition on global TK, a one-way ANOVA with *post hoc* Student-*t* test was performed. Differences in regional TK were examined with a one-way ANOVA for each Release condition and *post hoc* Student-*t* test comparing the region (superior, middle, inferior). For the effect of the planned correction with the pre-contoured rods, a paired Student-t test was performed on the rod RoC measurements before (pre) and after (post) rod reduction for each Release condition. The level of significance was set to *α* = 0.01.

## 3. Results

**Figure 4:**
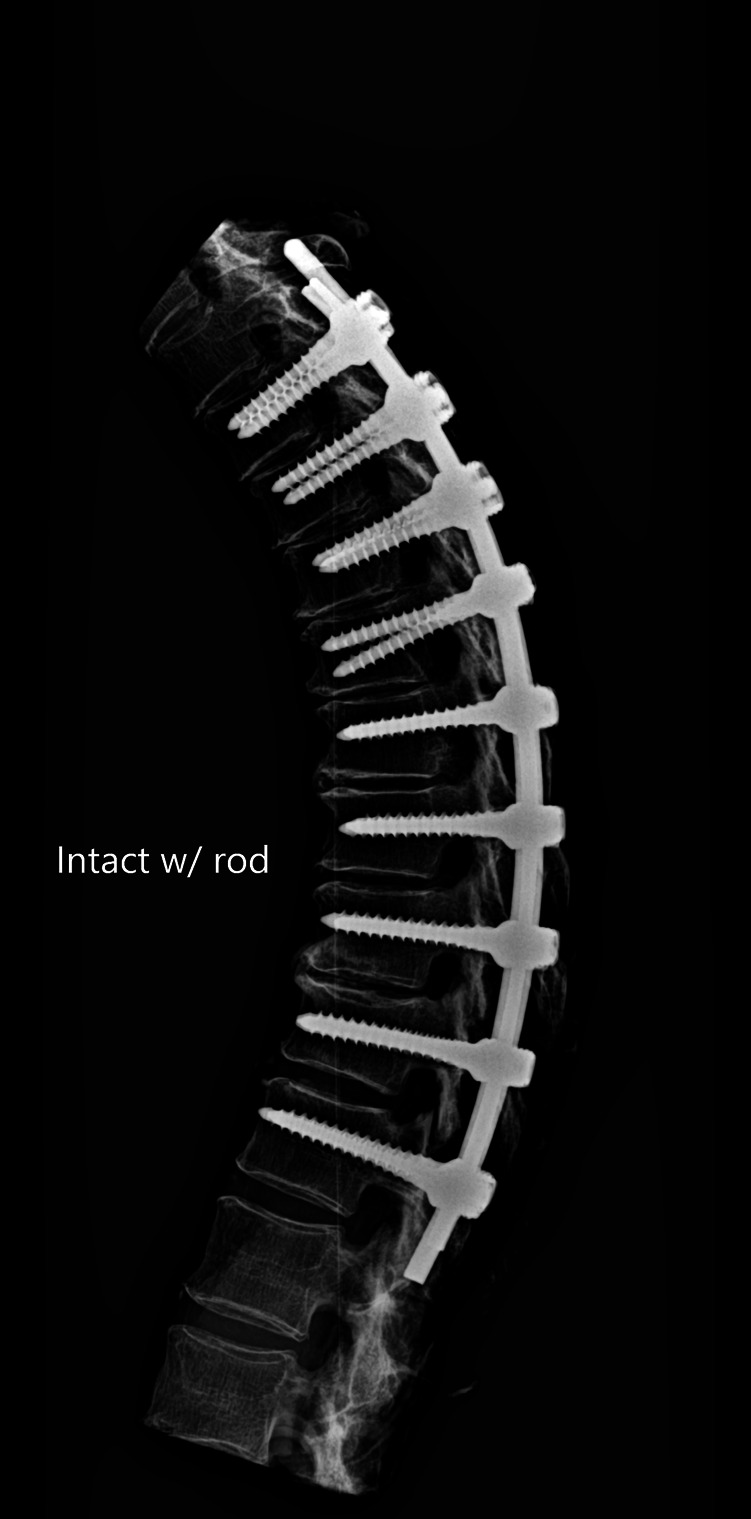
Lateral radiograph of sequential release of the thoracic spine with pre-contoured rods to induce thoracic kyphosis: Release 0—intact with over corrected bilateral rods.

**Figure 5:**
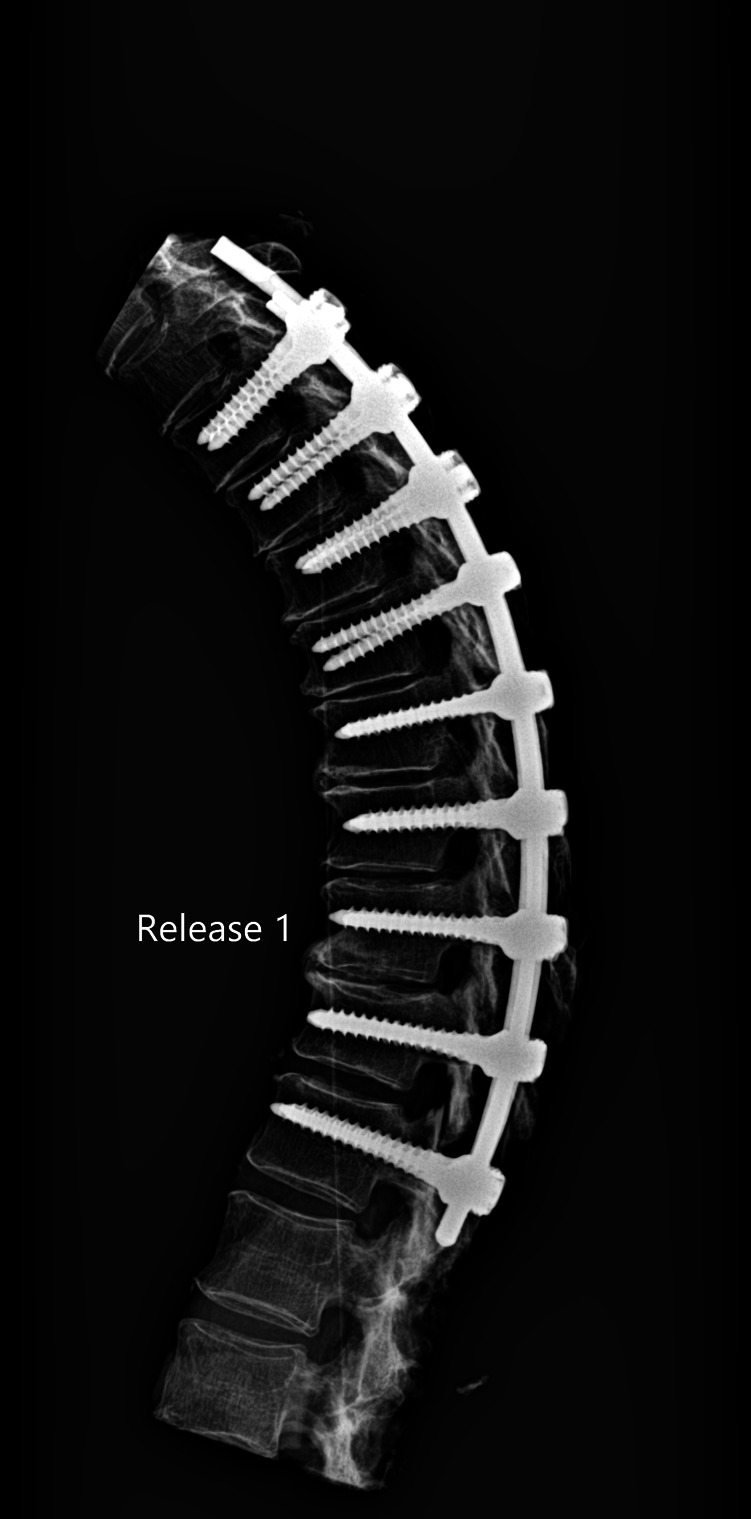
Lateral radiograph of sequential release of the thoracic spine with pre-contoured rods to induce thoracic kyphosis: Release 1—ISL transected.

**Figure 6:**
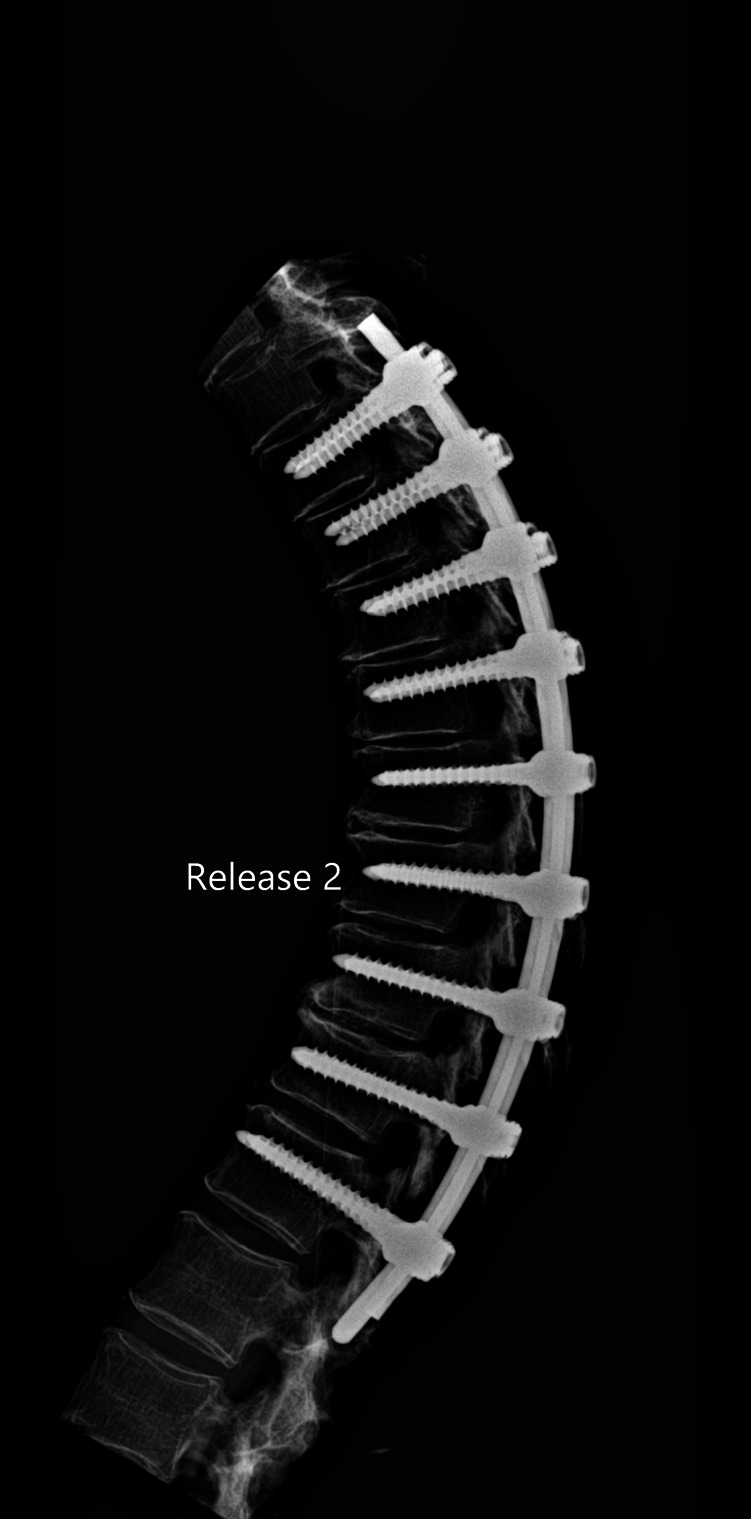
Lateral radiograph of sequential release of the thoracic spine with pre-contoured rods to induce thoracic kyphosis: Release 2—ligamentum flavum transected.

**Figure 7:**
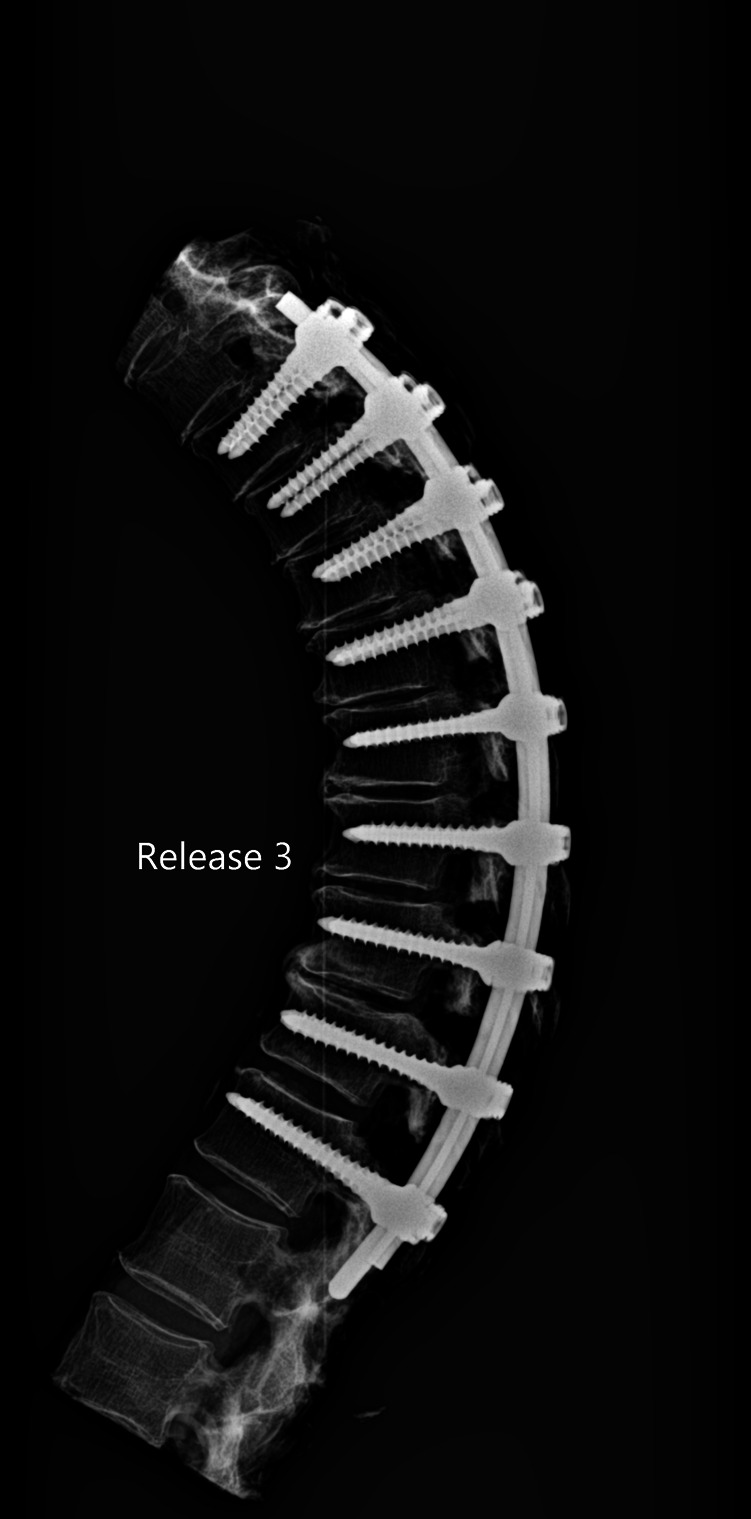
Lateral radiograph of sequential release of the thoracic spine with pre-contoured rods to induce thoracic kyphosis: Release 3—facet joints removed and consistent with a Ponte osteotomy.

**Figure 8:**
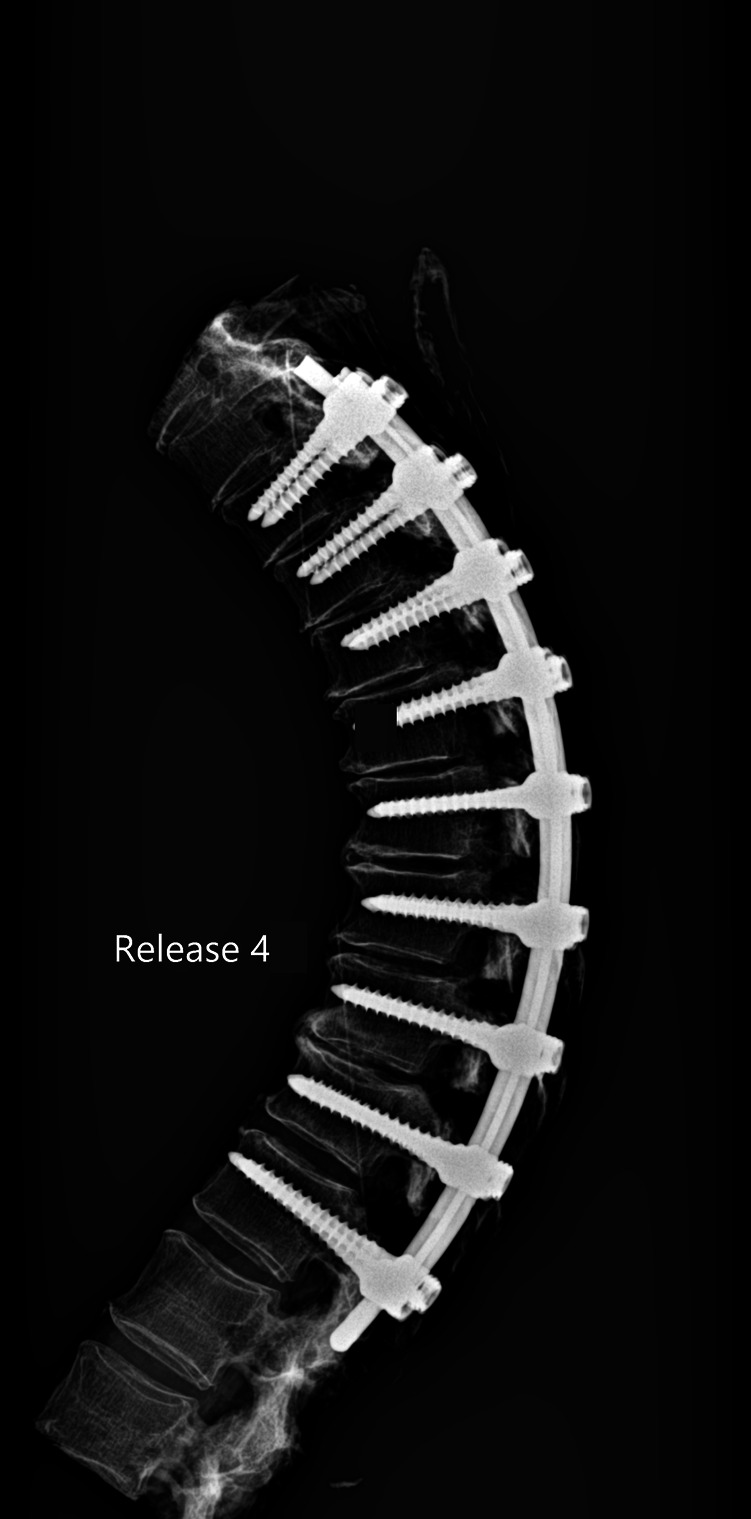
Lateral radiograph of sequential release of the thoracic spine with pre-contoured rods to induce thoracic kyphosis: Release 4—PLL transected.

**Figure 9:**
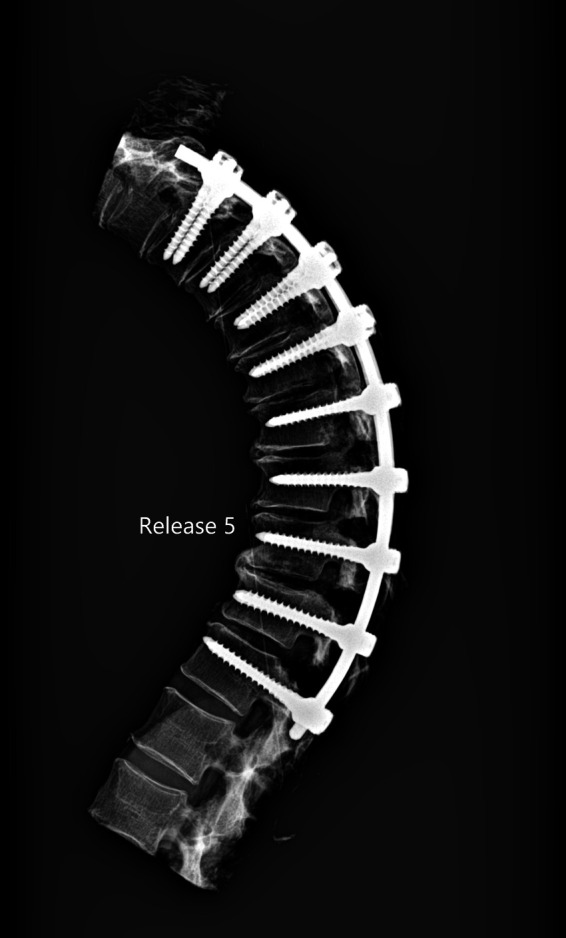
Lateral radiograph of sequential release of the thoracic spine with pre-contoured rods to induce thoracic kyphosis: Release 5—transforaminal discectomy.

The release procedures were thoroughly performed. There were no screw pull-outs following overcorrection and rod reduction for all the releases performed. An example of an instrumented thoracic spine in the various stages of release is shown in Figures 4–9.

**Figure 10:**
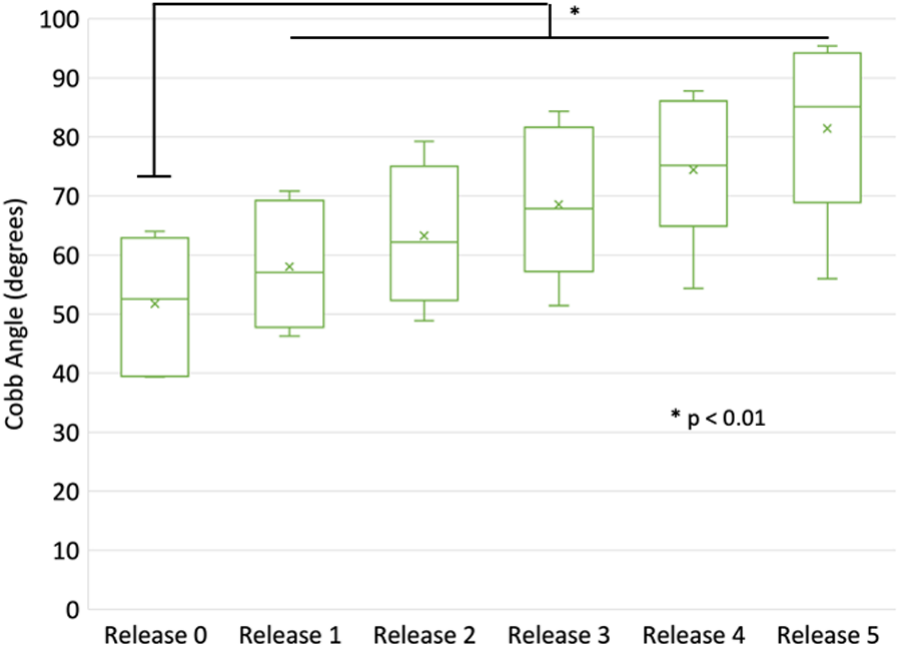
Comparison of global (T4–T12) thoracic kyphosis following the various stages of release and applied bilateral rods (over corrected and reduced).

The average intact global TK from T4–T12 was 38.0° (range 22.1°–51.4°) and TK significantly increased to 51.7° (range 39.4°–64.0°) with overcorrection and pre-contoured rods applied (Table 1, Release 0). The average initial RoC prior to placement and reduction for the Release 0 condition was 42.1° (range 28.5°–53.5°) and increased incrementally to an average final RoC of 55.4° (range 46.6°–64.0°) prior to placement and reduction for the Release 5 condition. Each surgical release resulted in a significant (*p* < 0.01) increase of 5°–7° of additional kyphosis compared to the intact status with the rods (Figure 10).

**Table 1:** Thoracic kyphosis measurements (T4–T12) for intact, uninstrumented thoracic spine and after each release condition with instrumented rods (degrees).

Specimen	Intact	Release 0	Release 1	Release 2	Release 3	Release 4	Release 5
1	33.2	39.4	46.3	48.8	51.4	54.4	56.0
2	38.9	64.0	68.7	79.2	84.4	90.4	93.8
3	22.1	39.4	48.3	53.4	59.1	69.7	78.9
4	51.4	61.4	64.2	68.7	73.6	84.2	91.3
5	44.8	62.6	70.8	73.6	80.7	85.5	95.4
6	37.5	43.7	50.0	55.6	62.1	66.9	73.2
Average	38.0	51.7	58.0	63.2	68.6	75.2	81.4

**Figure 11:**
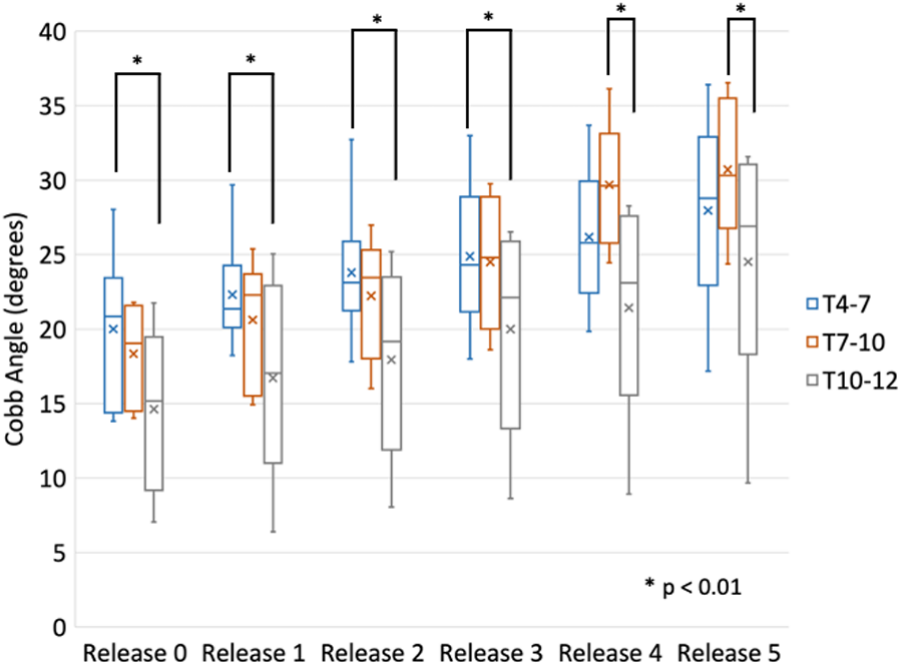
Comparison of regional (T4–T7, T7–10, T10–12) thoracic kyphosis following the various stages of release and applied bilateral rods (over corrected and reduced).

Regionally, kyphosis increased following successive surgical releases, which was approximately 1° at the superior region and approximately 2° at the middle and inferior regions for Releases 2–5 (Table 2). The largest increase occurred in the T7–10 region when the ISL was transected. There were significant differences (*p* < 0.01) between the regions T4–7 and T10–12 for the posterior releases (Release 2 and 3), and subsequent anterior releases (Release 4 and 5) displayed significant changes between the T7–10 and T10–12 regions (Figure 11).

**Table 2:** Average regional thoracic kyphosis measurements for the intact, uninstrumented thoracic spine and after each release condition with instrumented rods (degrees).

TK	Intact	Release 0	Release 1	Release 2	Release 3	Release 4	Release 5
Superior^a^	16.0	20.0	22.3	23.8	24.9	25.7	26.4
Middle^b^	12.2	18.3	20.6	22.2	24.5	27.2	30.7
Inferior^c^	10.6	14.6	16.7	17.9	20.0	22.0	24.5

^a^ Superior TK = upper endplate of T4 to upper endplate of T7.

^b^ Middle TK = upper endplate of T7 to upper endplate of T10.

^c^ Inferior TK = upper endplate of T10 to lower endplate of T12.

**Figure 12:**
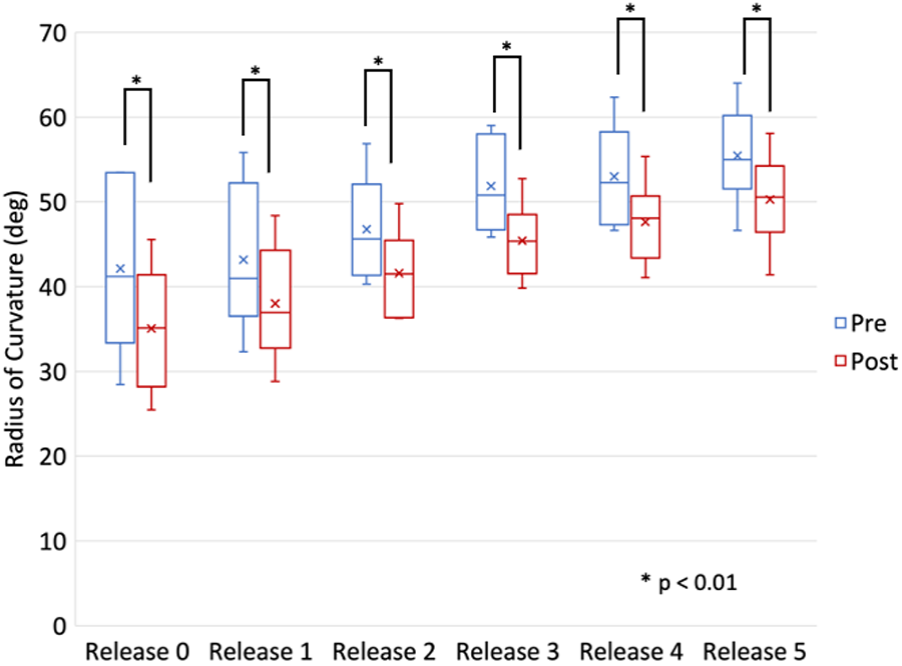
Comparison of the radius of curvature (RoC) of the bilateral rods pre- and post-instrumentation and reduction during various stages of release.

Comparing the radius of curvature of the pre-contoured rods before and after application showed a significant loss in rod curvature of 6° (*p* < 0.001) and this loss was independent of release type (Figure 12).

## 4. Discussion

Main thoracic curve scoliosis, which is defined as Lenke Type 1 scoliosis, is the most common type of adolescent idiopathic scoliosis. Surgeons have gradually recognized that the restoration of sagittal alignment of the main thoracic curve is also important in addition to the correction of the coronal plane during surgery (16, 17). In a Lenke Type 1 and 2 adolescent idiopathic scoliosis study, Hwang et al. (18) reported the sagittal alignment of the thoracic spine is related to that of the cervical spine when treated with surgery. The cervical spine may decompensate into significant kyphosis if postoperative TK is excessively decreased. Another study (19) concluded TK and lumbar lordosis (LL) both tend to decrease after posterior correction surgery; therefore LL-TK may be an important compensatory mechanism in maintaining sagittal balance. It is reasonable to deduce that a decrease of TK after surgical correction has the potential to increase the risk of related adverse consequences, such as, disc degeneration, flat back, sagittal imbalance, and a poorer quality of life. Additionally, thoracic hypokyphosis of less than 20° has been correlated with decreased pulmonary function.

Many factors may influence TK during surgery. The surgeon's experience, feature of the deformity, rod stiffness, the shape of the pre-countered rods, pedicle screw density, flexibility of the spine and release procedures all contribute to the corrective effort of the curve of the thoracic spine (20, 21). In this current *in vitro* biomechanical study, the focus was to examine TK and understand the effect of sequential releases and correction with symmetric, pre-contoured rods. This *in vitro cadaveric* model and study design had a number of limitations that could have influenced the results. The global and regional TK in the current *in vitro* study were similar to the TK described in adult, asymptomatic volunteers by Lafage et al. (22). All thoracolumbar specimens were screened and selected to ensure flexibility, however how the flexibility of the adult spine compares to the adolescent spine is unknown (23). Another limitation was that only symmetric, pre-contoured 5.5 mm titanium alloy rods were used and bilateral pedicle screws placement to induce the kyphosis of thoracic spine. The rods have a capacity of plastic deformation which may lead to loss of correction. Therefore, a novel rod-bender was utilized for symmetry and consistency to create a larger curve for the rods to induce the maximal thoracic kyphosis which was confirmed by the reduction in the rod RoC following application. Although Cobalt-chromium (CoCr) alloy rods are stiffer than Ti rods, the ability of deformity correction in adolescent idiopathic scoliosis (AIS) of the two rods is still controversial, and some studies have indicated that both rods can achieve sufficient correction in Lenke 1–3 types (24). Bowden et al. (25) showed CoCr rods had better ability of correction of TK compared with Ti rods in AIS from a meta-analysis. In our study, since one purpose was to measure the effectiveness of sequential posterior releasing procedures, we choose Ti rods because they are more flexible than CoCr rods which means the ability of TK correction by sequential posterior releasing procedures could be evaluated accurately. We placed bilateral pedicle screws at each level to achieve the highest screw density in order to provide better stabilization and corrective force (20). In our study, no screw pull-outs or loosening occurred in any of the six cadavers after the repeated correction procedures.

Lafage et al. has shown in asymptomatic individuals there was greater segmental kyphosis proximal to T7 than distal to T7 (22). One finding of the current *in vitro* study was the regional increase in TK following successive releases and over correction was proportionally greater in the regions distal to T7 (T7–T10, T10–12) in comparison to proximal (T4–7). This result could potentially be explained by the symmetry of the prepared over corrected rods. Clinically this may have significance in that rod curvature and over correction should account and target these regional kyphotic differences avoiding symmetric pre-contoured rods unless warranted.

The posterior release procedure included a ligamentous complex transection, bony resection, discectomy, and rib head resection. In a previous study, Cheng et al. (26) indicated that a posterior only release could provide a correction similar to an anterior–posterior combined approach. Rynearson et al. (27) performed an *in vitro* biomechanical analysis and concluded a wide posterior release of the thoracolumbar spine provided significant correction. However, they did not analyze the ability of each posterior release for sagittal alignment. Namikawa et al. (28) evaluated 24 patients with Lenke Type 1 or 2 AIS and found a concave rib head resection and convex transverse process resection as posterior release procedures could achieve satisfactory coronal and sagittal curve correction. In our study, we assessed the posterior release step by step and found each release resulted in 5°–7° of additional kyphosis, with the largest releases being ISL and PLL. Although an ISL transection procedure is easily accomplished, our results demonstrated that each release is additive. AIS patients are typically hypokyphotic and coronal correction maneuvers historically ignore or even exaggerate the hypokyphosis (8, 9). Doing an isolated ISL transection when attempting to correct the hypokyphosis in an AIS patient may not be adequate to achieve a substantial thoracic kyphosis restoration when only an average of 6.9° of kyphosis was produced by an ISL release in our study. Newton et al. confirmed that many AIS patients present with a preoperative thoracic kyphosis of less than 10° (8). Each release is additive in terms of imparted kyphosis. Adding a release of the ligamentum flavum and possibly the facet joints may be necessary to induce a more normal thoracic kyphosis of between 20° and 40°. We believe one reason for this observation was the stability of thoracic spine region which was enhanced by the rib cage. Another reason may be the space of the spinous process at thoracic region was small from an anatomic view, which means the range of motion could not change significantly. Therefore, we recommend doing an ISL transection and ligamentum flavum transection as posterior releases in order to gain a satisfactory sagittal curve in hypokyphotic patients with AIS. A Ponte osteotomy, PLL transection and discectomy as subsequent releases resulted in significant (*p* < 0.05) increases in correction compared to the intact condition with rod correction. However, these procedures are more aggressive and may potentially lead to more blood loss and higher neurological deficit risks. A multilevel Ponte osteotomy has been shown to be useful in restoring TK with AIS (29). In recent years, three-column osteotomy including PLL transection and discectomy has been used for AIS as well, especially in revision cases (30). However, in the current *in vitro* study, transection of the ISL and ligamentum flavum was enough to create thoracic kyphosis in AIS, so we do not recommend surgeons do Releases 3–5 routinely, although we evaluated the ability of TK correction of Releases 3–5.

The purpose of this cadaver study was to demonstrate the relative contributions of the intrinsic bony and ligamentous structures to the sagittal stiffness that must be overcome by a posterior based screw and rod instrumentation system that would typically be used to correct an idiopathic scoliosis. These deformities are often hypokyphotic and the restoration of more normal thoracic kyphosis is arguably important. The practicality and safety of releasing the posterior longitudinal ligament and the disc is limited but was completed in this study to attempt to demonstrate their relative contributions compared to more traditional releases of the posterior ligaments and facets. In that way, we believe the flexible spine specimens in our study have not hindered our results.

## 5. Conclusions

Increased kyphosis was created in the thoracic spine using pre-contoured rods. Subsequent posterior releases provided a substantial and meaningful change in the ability to induce additional kyphosis. Although each posterior release was effective for induction of TK (ISL and PLL having the largest increases), an ISL transection alone may not provide enough kyphosis to bring a hypokyphotic AIS spine into a more normal kyphosis as an average of 6.9 degrees of kyphosis was induced in our study specimens. More than one release was necessary to induce a clinical change in TK compared to the overcorrection accomplished on the intact thoracic spine with instrumentation. Reductions in radius of curvature of the titanium alloy rods consistently occurred following attempted over correction with rod reduction, even though the thoracic spine was sequentially destabilized with each release.

## Data Availability

The raw data supporting the conclusions of this article will be made available by the authors, without undue reservation.
